# Berberine Mediates the Production of Butyrate to Ameliorate Cerebral Ischemia via the Gut Microbiota in Mice

**DOI:** 10.3390/nu16010009

**Published:** 2023-12-19

**Authors:** Huijie Duan, Junya Hu, Yang Deng, Junqing Zou, Wangli Ding, Qiang Peng, Rui Duan, Jianguo Sun, Junrong Zhu

**Affiliations:** 1 Department of Pharmacy, Nanjing First Hospital, China Pharmaceutical University, Nanjing 210006, China; dhjsdcpu@163.com (H.D.); cpu_dy1210@163.com (Y.D.); dwl806267@163.com (W.D.); 2 Department of Pharmacy, Nanjing First Hospital, Nanjing Medical University, Nanjing 210006, China; zjq424@stu.njmu.edu.cn; 3 Department of Neurology, Nanjing First Hospital, Nanjing 210006, China; pengqiangkite@163.com; 4 Key Lab of Drug Metabolism and Pharmacokinetics, State Key Laboratory of Natural Medicines, China Pharmaceutical University, Nanjing 210009, China

**Keywords:** stroke, gut microbiota, butyric acid, microglia, astrocytes, NLRP3

## Abstract

Ischemic stroke (IS) is a vascular disease group concomitant with high morbidity and mortality. Berberine is a bioactive substance and it has been known to improve stroke, but its mechanism is yet to be proven. Mice were fed with BBR for 14 days. Then, the mice were made into MCAO/R models. Neurological score, infarct volume, neuronal damage and markers associated with inflammation were detected. We tested the changes in intestinal flora in model mice after BBR administration using 16SrRNA sequencing. Chromatography–mass spectrometry was used to detect butyrate chemically. Tissue immunofluorescence was used to detect the changes in the microglia and astroglia in the mice brains. Our findings suggest that berberine improves stroke outcomes by modulating the gut microbiota. Specifically, after MCAO/R mice were given berberine, the beneficial bacteria producing butyric acid increased significantly, and the mice also had significantly higher levels of butyric acid. The administration of butyric acid and an inhibitor of butyric acid synthesis, heptanoyl-CoA, showed that butyric acid improved the stroke outcomes in the model mice. In addition, butyric acid could inhibit the activation of the microglia and astrocytes in the brains of model mice, thereby inhibiting the generation of pro-inflammatory factors IL-6, IL-1β and TNF-α as well as improving stroke outcomes. Our results suggest that berberine may improve stroke outcomes by modulating the gut flora to increase the abundance of butyric acid. These findings elucidate the mechanisms by which berberine improves stroke outcomes and provide some basis for clinical treatment.

## 1. Introduction

Ischemic stroke (IS) secondary to stenosis or the occlusion of cerebral blood-supplying arteries is an essential risk factor for disability and death in the elderly; in the meantime, the age of onset of ischemic stroke has been gradually decreasing to a younger age in recent years [1,2]. The existing therapy for it is mainly thrombolytic agents (such as rt-PA), but this still has restrictions like poor safety [3]. Therefore, new strategies for the treatment of IS are yet to be pioneered.

The gut microbiota comprise a series of symbiotic microorganisms primarily located in the body’s gut. There is a two-way communication passage connecting the nervous system to the intestines whose name is the brain–gut axis [4]. Increasing studies have found that the processes of neurological diseases are accompanied by the regulation of gut microbiota, including in neurological diseases ranging from Parkinson’s disease (PD) [5] to Alzheimer’s disease (AD) [6]. The gut microbiota and the nervous system regulate the homeostasis of the host via direct or indirect signaling. For example, butyrate, a metabolite of the gut microbiota, directly mediates the gut–brain axis in AD model mice to reduce microglia-associated neuroinflammation [7]. Similarly, several bodies of research have found that the gut microbiota were engaged in ischemic stroke outcomes. For example, intestinal symbiotic dysregulation exacerbates IS damage by regulating effector T-cells [8]. Equally, fecal microbial transplantation was reported to improve cognitive impairment in IS mice [9]. However, the specific regulatory mechanisms of the gut microbiota’s involvement in ischemic stroke remain to be determined.

Butyric acid, as a short-chain fatty acid (SCFA), is a lipid generated by the gut microbiota via the fermentation of dietary fibers [10]. Increasingly, the evidence illustrated that the gut microbiota with their SCFA metabolites (especially butyric acid) seem to be pivotal signaling elements for the brain–gut axis. A study showed that butyrate administration improved toxicity and had anti-neuroinflammatory effects in mice on a high-fat diet [11]. Meanwhile, butyrate administration is proved to exert beneficial effects by reducing neuronal mitochondrial damage and oxidative stress in a mouse model of epilepsy [12]. Remarkably, a study identified the therapeutic role of the transplantation of butyric acid-rich fecal flora into a rat model of IS [13]. Recent studies have found that the amount of butyrate-producing gut bacteria and the concentration of butyrate are strongly correlated with stroke outcomes [14,15]. However, the mechanism of butyrate, a metabolite of the gut microbiota, as a therapeutic target for IS needs to be further explored.

Berberine (BBR) is extracted from Coptis, a traditional Chinese medicine, and can also be synthesized artificially [16]. At present, the benefits of berberine in a multitude of neurological disorders have been discussed. For example, BBR targeted the NLRP3 inflammasome to alleviate Parkinson’s-related pathologies in a mice model [17], and berberine targeting mitochondrial autophagy alleviated Alzheimer’s-like pathology in mice [18]. Moreover, although our previous studies have proved that berberine has a neuroprotective effect in IS [19,20,21], berberine cannot be absorbed into circulation in large quantities because of its low solubility and large molecular weight, so it may remain in the intestines and interact with the gut microbiota [22]. Berberine has been reported to play a prominent role in diabetes and hyperlipidemia by regulating the abundance of the gut microbiota and their metabolite production [23,24]. However, whether berberine exerts neuroprotective effects in IS by influencing the production of gut–microbiota-related metabolites is unclear.

Herein, we contemplate on employing 16S rRNA gene sequencing together with fecal microbial transplantation to elucidate the regulation of the gut microbiota and their metabolite butyrate using berberine in MCAO/R mice. This research suggested that berberine-mediated neuroprotection may be dependent on the gut-microbiota-related butyrate metabolism. This study also provides an experimental rationale for the clinical use of berberine during therapy for IS.

## 2. Materials and Methods

### 2.1. Animals and Administration

C57BL/6J mice (8–10 weeks, male, 25–30 g) were obtained from the Model Animal Research Center of Nanjing University (Nanjing, China), and the mice experiments and procedures were approved by the Ethics Committee of Nanjing First Hospital (Approval Number of Ethics Committee: DWSY-23063367). The mice were placed in a suitable environment (including suitable temperature and humidity control and with a water and food supply). 

For the administration of sodium butyrate, different concentrations of sodium butyrate (Aladdin Inc., Shanghai, China) dissolved in normal saline were administered via gavage for 14 sequential days. For the administration of heptanoyl-CoA, the heptanoyl-CoA (Sigma-Aldrich, Oakville, ON, Canada) was dissolved in normal saline, and the dose was 0.4 mg/kg and administered rectally [25,26].

### 2.2. Fecal Microbial Transplantation (FMT)

Firstly, mice were given berberine (purity 98%, BP1108, Sigma, St. Louis, MO, USA) solution 50 mg/kg (dissolved in 0.5% CMC-Na) or normal saline only for 14 consecutive days. The choice of BBR dose and route of administration was based on our previous studies [20,21]. The feces were collected, immediately suspended with sterile PBS, and the dissolved feces were centrifuged. The collected suspension was filtered by a 70 μm sterile filter. Mice pretreated with antibiotics metronidazole (1 g/L, Aladdin, Shanghai, China), vancomycin (0.5 g/L, Aladdin, Shanghai, China), ciprofloxacin (0.2 g/L, Aladdin, Shanghai, China) and neomycin (1 g/L, Aladdin, Shanghai, China) dissolved in the drink water for 14 days were given the fecal suspension by intragastric administration. Each mouse was given 200 μL of fecal suspension per day, and the MCAO/R model was constructed after 7 days of continuous intragastric administration [27,28,29].

### 2.3. Animal Model of MCAO/R

The method of Longa et al. was used for the MCAO/R operation, and some improvements were made [30]. The mice were anesthetized by isoflurane (2%, Abbott Laboratories, Abbott Park, MD, USA). The procedure of setting up is as follows: a median incision in the neck, a blunt separation of the neck muscles, and the exposure of the right common cervical, external cervical (ECA), and internal carotid (ICA) arteries under an operating microscope; Ligate ECA opposite End and cut the proximal end, insert a filament (Cinontech, Guangzhou, China) into the ECA along the ICA into the middle cerebral artery. After 1 h of ischemia, the filament was removed to restore blood perfusion.

### 2.4. Neurological Score Determination 

Neurological impairment score was put at 24 h after MCAO/R. The specific method is based on the five-point scale (0–4) described by Longa et al. The scoring criteria are as follows: 0, no deficit, normal walking; 1, mild deficit, fail to stretch forepaw; 2, moderate deficit, circling to the contralateral side; 3, severe deficit, falling to the contralateral side; 4, no spontaneous moving.

### 2.5. TTC Staining

The brain tissue was frozen at −20 °C for 20 min, and coronal sections were made for about 2 mm. The prepared brain tissue was added in 0.5% TTC (Sigma-Aldrich, St. Louis, MO, USA); then, it was strictly sealed in a 37 °C constant temperature water bath, dyed for 30 min away from light, and turned the brain sheet every 10 min to make it evenly contact with the dye. TTC staining can stain normal brain tissue reddish–brown and cause ischemic infarction to appear pale. They were placed neatly on a black background and photographed. The percentage of infarct volume in the whole brain volume was calculated by ImageJ (National Institutes of Health, Bethesda, MD, USA).

### 2.6. Hematoxylin and Eosin Staining

The brains of MCAO/R mice were embedded in paraffin after cardiac perfusion and extraction. The experimental steps of HE staining are as follows. First, we put the slices into xylene I, xylene II, anhydrous ethanol I, anhydrous ethanol II, and 75% alcohol in turn. Then, we put the slices in the hematoxylin dye solution (#G1001-100ML, Servicebio, Wuhan, China) and differentiated them. The sections were dehydrated with gradient alcohol and then stained with eosin solution (#G1004-100ML, Servicebio, Wuhan, China). Afterwards, the slices were successively placed into anhydrous ethanol I, anhydrous ethanol II, anhydrous ethanol III, dimethyl I, and xylene II in turn. The morphological changes were observed and photographed with an Olympus microscope (Olympus™, Tokyo, Japan).

### 2.7. Nissl Staining

The brains of MCAO/R model mice were sliced and sealed with paraffin after cardiac perfusion; then, they were observed and photographed with an Olympus microscope (Olympus™, Tokyo, Japan) after Nissl staining (#G1036-100ML, Servicebio, Wuhan, China).

### 2.8. Pyrosequencing Using 16S rDNA Amplicon

Firstly, the total genomic DNA was extracted by the CTAB/SDS method. After quantifying DNA by 1% agarose gel, the sequence was amplified by a series of primers. 16S rRNA genes were amplified using the specific primer. PCR products purified by a GeneJET gel extraction kit (Thermo Scientific, Waltham, MA, USA) were selected before sequencing. The library was then prepared and sequenced using the Illumina TruSeq DNA PCR-Free Library Preparation Kit (Illumina, San Diego, CA, USA) as recommended by the manufacturer. Library quality was assessed using the Qubit@2.0 fluorometer (Thermo Scientific, Waltham, MA, USA) and the Agilent Bioanalyzer 2100 system. Finally, the library was sequenced on Illumina NovaSeq PE250 (Illumina, San Diego, CA, USA) platform. Alpha-diversity, beta-diversity, and bacterial community composition were analyzed by using the QIIME tool. Linear discriminant analysis (LDA) effect size (LEfSe) was performed with an LDA score > 2.0.

### 2.9. Butyrate Quantification

Fecal samples were taken for colon contents. The blood samples were centrifuged through cardiac puncture blood at 4 °C, 3000 rpm and plasma were taken. The brain samples were homogenized in a homogenizer with PBS after brain extraction, and all samples were immediately frozen on dry ice. The sample was dissolved in ddH_2_O and mixed with 2-methylbutyric acid. After centrifugation, the supernatant was derived using an isopropanol–pyridine solution and a platelet cytotoxic factor solution and then extracted and analyzed using N-hexane. The sample was analyzed using the Agilent 7890A/5975C gas chromatography–mass spectrometry system. The specific method follows: nitrogen is used as the carrier gas, and the flow rate is controlled to 1 mL/min. Set the column temperature starting temperature to 100 °C and maintain for 0.5 min. Then, the temperature is raised to 180 °C at a rate of 8 °C/min for 1 min and then to 200 °C at a speed of 10 °C/min for 5 min. The temperature of the detector is controlled to 240 °C, the temperature of the inlet is set to 200 °C, and the sample volume is 1 μL. The detection time of each specimen was 5 min.

### 2.10. Butyryl-CoA Acetate-CoA Transferase (BUT) Assay

Butyryl-CoA acetate-CoA transferase (BUT) activity was determined with reference to relevant literature [31]. The activity of butyryl-CoA: acetate-CoA transferase (BUT) was determined by detecting the formation of acetyl-CoA using acetic acid and butyrate as substrates. Acetyl-CoA concentration was determined using the Acetyl-CoA assay kit (Jiancheng Bioengineering Institute, Nanjing, China) according to the manufacturer’s instructions.

### 2.11. Western Blot Analysis

The extraction of total proteins from brain tissues was completed with RIPA buffer (Cell Signaling Technology, Boston, MA, USA). A BCA Protein Quantification Kit (Key GEN Biotech, Nanjing, China) was used to measure the protein concentration. Protein samples of the same amount (30 μg) were separated by SDS-PAGE and transferred to PVDF membranes (Millipore, Billerica, MA, USA). The membranes were sealed with 5% milk and combined with anti-NLRP3 (1:1000, #15101, Cell Signaling Technology, Boston, MA, USA), anti-SYP (1:1000, #ab52636, Abcam, Cambridge, UK), anti-PSD95 (1:5000, #ab2723, Abcam, Cambridge, UK), anti-β-actin (1:1000, #4970, Cell Signaling Technology, Boston, MA, USA), anti-GFAP (1:1000, #ab68428, Abcam, Cambridge, UK), anti-Iba-1 (1:1000, #ab178846, Abcam, Cambridge, UK) at 4 °C overnight. Next, we used the corresponding secondary antibody to incubate the film at room temperature for 2 h. Immune complexes were assessed with an ECL kit (Thermo Scientific, Waltham, MA, USA), and the results were quantified by ImageJ.

### 2.12. Real-Time Reverse Transcription-Quantitative PCR (RT-qPCR)

In order to detect the mRNA expression levels of related inflammatory factors, RT-qPCR was performed according to the description [32]. The following primers were used: β-actin: forward TGAGCTGCGTTTTACACCCT, reverse GCCTTCACCGTTCCAGTTTT; IL-1β: forward CACCTCTCAAGCAGAGCACAG, reverse GGGTTGCATGGTGAAGTCAAC; IL-6: forward GCTACCAAACTGGATATAATCAGGA, reverse CCAGGTAGCTATGGTACTCCAGAA; TNF-α: forward GACCCTCACACTCAGATCATCTTCT, reverse CCTCCACTTGGTGGTTTGCT.

### 2.13. ELISA

The brain homogenates’s IL-6 (M6000B, R&D Systems, Inc., Minneapolis, MN, USA), TNF-α (MTA00B, R&D Systems, Inc., Minneapolis, MN, USA) and IL-1β (MLB00C, R&D Systems, Inc., Minneapolis, MN, USA) levels were determined using a corresponding ELISA kit.

### 2.14. Immunofluorescence

The hearts of the mice were perfused. The brains were sliced open and then embedded in paraffin. After the dewaxing and rehydration of the conventional paraffin section, the anti-priming was performed by an elevated water bath. We placed the slices on a dyeing rack, in a little enameling jar filled with 10 mm·L^−1^ lemon-sodium lemon-buffer (pH 6.0), and placed the enameling jar in a large boiling cup filled with a certain amount of fresh water. The electric furnace was heated and boiled, until the porcelain cylinder reached 92~98 °C for 15 min, and then the end was removed from the electric furnace and slowly cooled to room temperature. The distilled water was washed 2×, and the PBST was then immersed for 5 min. Then, it was fixed with 4% polymethylaldehyde for 10 min. We washed again with TBST for 2 × 5 min; the non-specific reaction sites were closed after incubation with 10% BSA for 1 h. We added anti-GFAP (1:400, #ab4648, Abcam, Cambridge, UK) or anti-Iba-1 (1:400, #ab178846, Abcam, Cambridge, UK), incubated it overnight at 4 °C, and then washed it with PBST for 3 × 10 min. The goat anti-rabbit IgG H&L (Alexa Fluor^®^ 488) (1:200, #ab150113, Abcam, Cambridge, UK) or goat anti-rabbit IgG H&L (Alexa Fluor^®^ 594) (1:200, #ab150080, Abcam, Cambridge, UK) was added and incubated at 37 °C for 1 h. We washed with PBST for 3 × 10 min. After washing, we stained the specimen with DAPI (Sigma, St. Louis, MO, USA) for 15 min. Finally, we photographed the samples with a laser scanning confocal microscope (Zeiss, Oberkochen, Germany).

### 2.15. Statistical Analyses

All data were presented as mean ± standard deviation (SD). GraphPad prism 8.0 (GraphPad Software, Inc., La Jolla, CA, USA) was utilized for following the analysis of experimental results. An unpaired *t*-test was used for a comparison between two groups, and one-way ANOVA was used for comparison among multiple groups. When *p* < 0.05, differences were considered significant.

## 3. Results

### 3.1. BBR Improves Stroke Outcomes by Regulating Gut Flora

**Figure 1:**
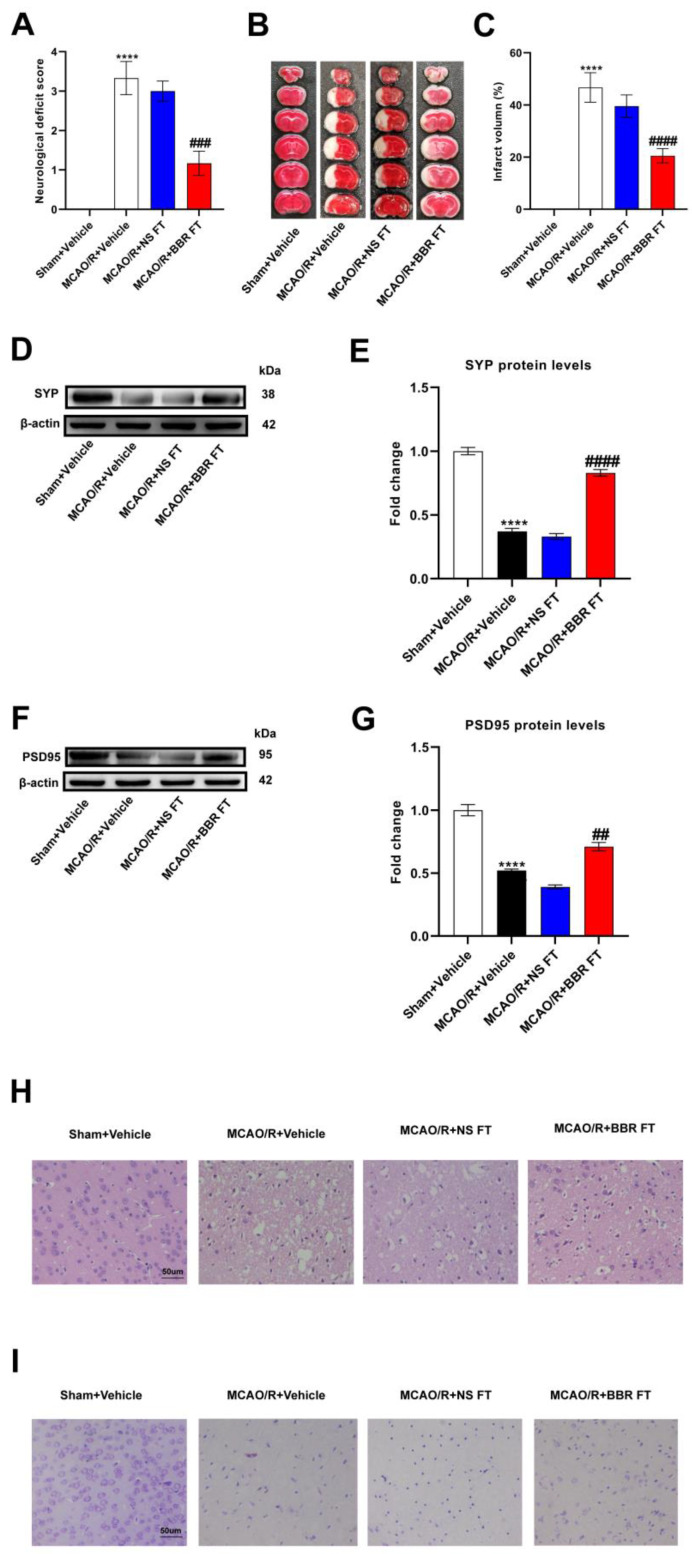
BBR improves stroke outcomes by regulating gut microbiota. (**A**) Neurological scores were measured 24 h after MCAO/R (*n* = 18). (**B**) TTC staining was performed 24 h after MCAO/R (*n* = 6). (**C**) The percentage of infarct volume to Sham value was analyzed 24 h after MCAO/R (*n* = 6). (**D**,**E**) Western blot and quantitative analysis showed the expression of SYP in mice brain (*n* = 6). (**F**,**G**) Western blot and quantitative analysis showed the expression of PSD95 in mice brain (*n* = 6). (**H**,**I**) Representative images of ischemic cerebral tissue with HE staining or Nissl staining (*n* = 6). Scale bar, 50 µm. Results were presented as mean ± SD. **** *p* < 0.0001 versus the Sham + Vehicle group; ^##^ *p* < 0.01, ^###^ *p* < 0.001, ^####^ *p* < 0.0001 versus the MCAO/R + Vehicle group.

To investigate whether berberine improved IS outcomes in connection with gut flora, we transplanted the intestinal flora of mice pretreated with berberine into each group of mice for the experiment. The MCAO/R + Vehicle mice had a significant increase in the neurological deficit scores and infarct volume versus the Sham group, indicating neurological damage, while mice transplanted with BBR-induced gut flora had relatively lower scores and infarct volume. However, there were no significant differences in the scores and infarct volume of the mice transplanted with the normal saline-induced gut flora versus the MCAO/R + vehicle group (Figure 1A–C). In addition, Western blot proved that the expressions of two synaptic-associated proteins (PSD95 and SYP) in the MCAO/R + Vehicle group and MCAO/R + NS group were both obviously lower than those in the Sham + Vehicle group, whereas the proteins’ expressions in the MCAO/R + BBR FT group were significantly higher compared to the above two groups (Figure 1D–G). The HE and Nissl staining assay showed that the neurons in the Sham + Vehicle group are normal, rounded and intact, with intact nuclei and tissue structure and numerous Nissl bodies, but both in the MCAO/R + Vehicle group and MCAO/R + NS FT group, we observed karyotyping, vacuolization, neuronal body atrophy, and markedly decreased Nissl volume. On the contrary, the MCAO/R + BBR FT mice showed a relative improvement in the structure of cavities and nuclei in neurons (Figure 1H,I). Taken together, these results demonstrate that the transplantation of a BBR-pretreated gut microbiome significantly improves stroke outcomes in mice compared to the transplantation of a saline-treated gut microbiome, suggesting that BBR modulates the gut microbiome to improve stroke outcomes in mice.

### 3.2. BBR Administration Increases the Abundance of Butyric-Producing Bacteria in MCAO/R Mice

**Figure 2:**
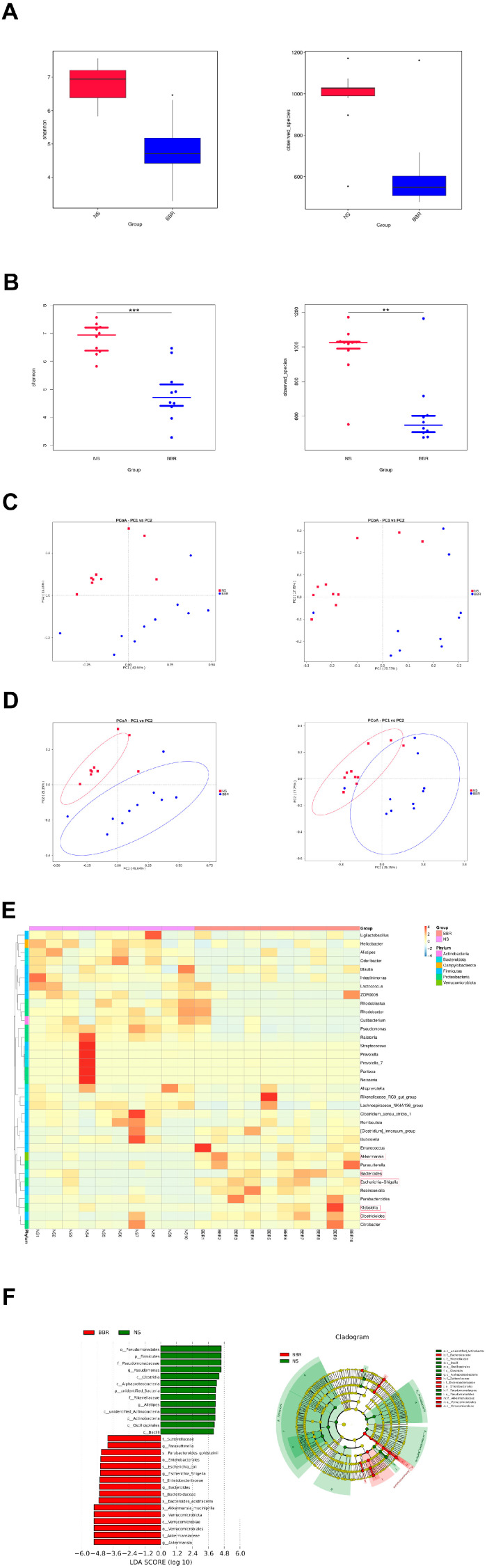
BBR administration increases the abundance of butyric−producing bacteria in mice. (**A**,**B**) Alpha diversity is expressed through the Shannon diversity index (*n* = 10). (**C**) The β−diversity (PCoA) of gut microbiota in each group (*n* = 10). (**D**) The relative abundance and the sample similarity of microbiota in each sample as well as the related heatmap clustering analysis (*n* = 10). (**E**,**F**) Significantly discriminative taxa between each group detected by linear discriminant analysis effect size (LDA effect size) and the taxonomic cladogram (*n* = 10). Data were presented as mean ± SD. ** *p* < 0.01, *** *p* < 0.001 versus the NS group.

To further elucidate how berberine specifically modulates the gut flora of MCAO/R mice, mice were given 50 mg/kg BBR or normal saline for 14 days in advance, and then the two groups underwent MCAO/R surgery. Three days later, fresh feces were sequenced with 16Sr DNA, and the abundance and components of the gut flora were calculated. After analysis, the findings showed that BBR as a natural antibacterial agent could significantly suppress the abundance and variety of gut microbiota in the MCAO/R group (Figure 2A,B). Meanwhile, the following PCoA analysis revealed that significant differences in the gut microbiome were observed between mice modeled after BBR treatment and those modeled after saline treatment (Figure 2C,D). Heatmap analysis showed that BBR pretreatment increased the production of butyric acid in most bacterial genera in the model mice, including Lachnoclostridium, Clostridioides, Escherichia–Shigella, Bacteroides, Akkermansia, Klebsiella; whereas in MCAO/R mice, it increased in Odoribacter, Alistipes, Clostridium_sensu_stricto_1 and Helicobacter, and they are associated with the development of inflammation [27] (Figure 2E). LefSe was utilized to detect significant abundance differences between the two groups. The predominant floras identified in the MCAO/R group included Pseudomonas, Clostridia, Alistipes, and Bacilli, which were mostly pertaining to inflammation. The predominant bacterial communities of the MCAO/R + BBR group were Akkermansia, Escherichia–Shigella, Bacteroides and Parasutterella; all of them could be metabolized to produce short-chain fatty acids (Figure 2F). Overall, these results demonstrate the ability of BBR to modulate the gut microbiome in MCAO/R mice, increasing beneficial bacteria-producing butyrate and decreasing bacteria associated with inflammation.

### 3.3. BBR Administration Increases the Content of Butyric Acid in MCAO/R Mice

**Figure 3:**
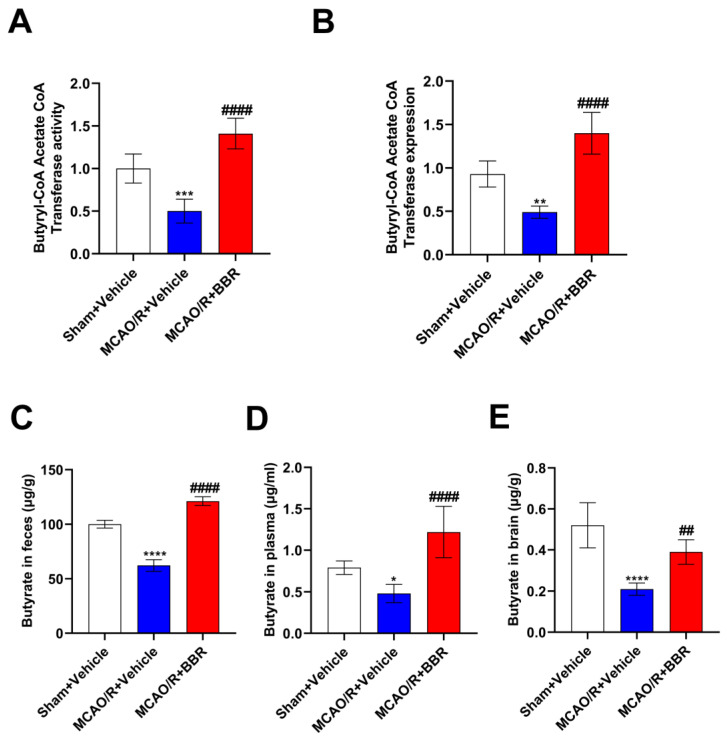
BBR administration increases the content of butyric acid in MCAO/R mice. (**A**,**B**) The activity and level of butyryl-CoA:acetate-CoA transferase in every group (*n* = 6). (**C**–**E**) The content of butyric acid in feces, plasma and brain of mice was determined by GC-MS (*n* = 6). Data are shown as mean ± SD. * *p* < 0.05, ** *p* < 0.01, *** *p* < 0.001, **** *p* < 0.0001 versus the Sham + Vehicle group; ^##^ *p* < 0.01, ^####^ *p* < 0.0001 versus the MCAO/R + Vehicle group.

BBR could upregulate the abundance of butyric acid-producing bacteria in the intestine of MCAO/R mice; thus, we further examined the content of butyric acid and the key enzyme in butyric acid synthesis in the mice after BBR administration: the activity of synthesis of butyryl-CoA acetate-CoA transferase (BUT). Compared with the corresponding controls, the expression and activity of BUT in MCAO/R mice decreased, while berberine administration could strikingly increase the activity and expression of BUT (Figure 3A,B). We further measured the butyrate content in the feces, plasma, and brain in each group of mice by GC-MS. The results showed that the content of butyrate in the body of MCAO/R mice decreased, while BBR administration could dramatically increase the content of butyrate in the body of MCAO/R mice (Figure 3C–E). To sum up, the above results indicated that the butyrate content in MCAO/R mice was significantly decreased and BBR could promote the synthesis of butyrate in MCAO/R mice.

### 3.4. Butyric Acid Exerts a Neuroprotective Effect in the MCAO/R Mice

**Figure 4:**
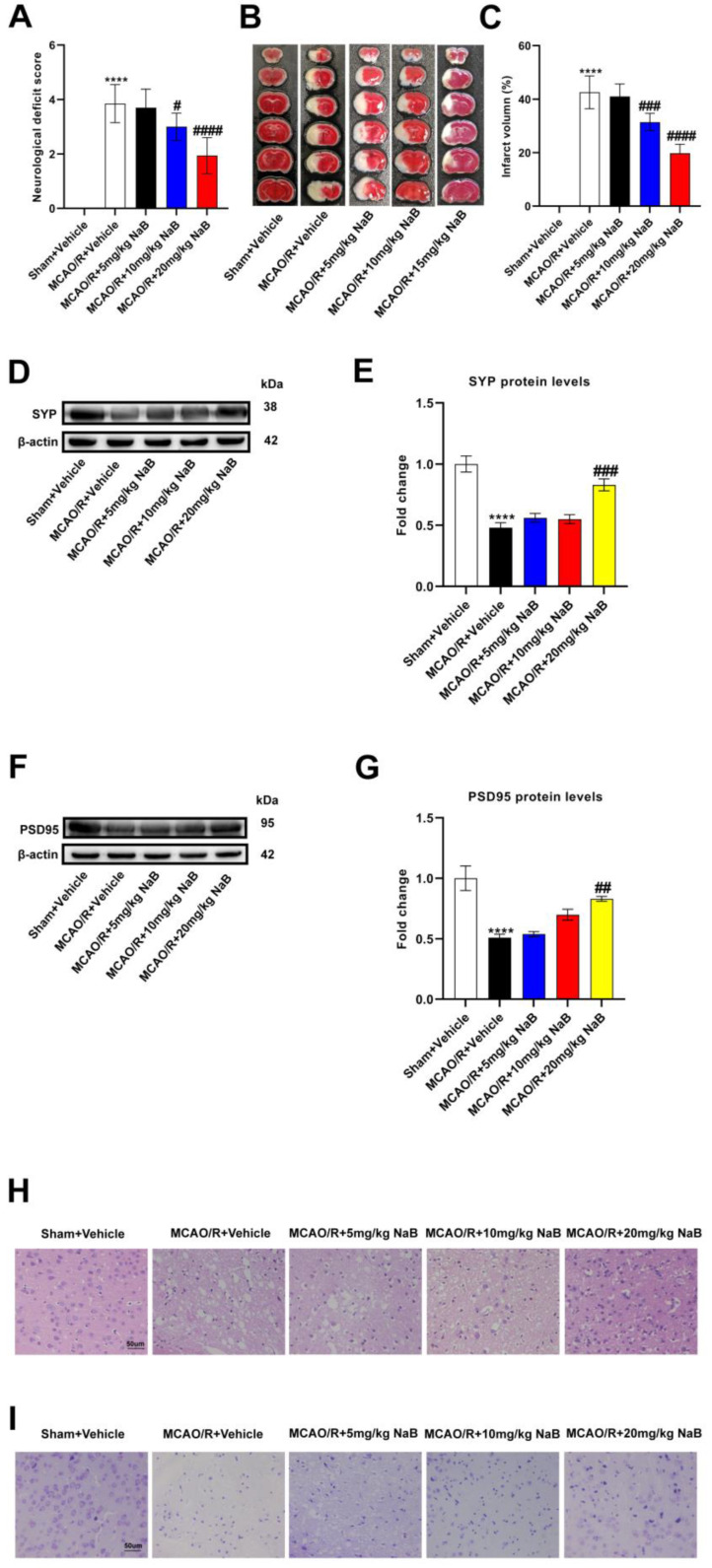
Butyric acid plays a protective role in the MCAO/R mice brain. (**A**) Neurological scores were measured 24 h after MCAO/R (*n* = 18). (**B**) TTC staining was performed 24 h after MCAO/R (*n* = 6). (**C**) The percentage of infarct volume to Sham value was analyzed 24 h after MCAO/R (*n* = 6). (**D**,**E**) Western blot and quantitative analysis showed the expression of SYP in mice brain (*n* = 6). (**F**,**G**) Western blot and quantitative analysis showed the expression of PSD95 in mice brain (*n* = 6). (**H**,**I**) Representative images of ischemic cerebral tissue with HE staining or Nissl staining (*n* = 6). Scale bar, 50 µm. Results are shown as mean ± SD. **** *p* < 0.0001 versus the Sham + Vehicle group; ^#^ *p* < 0.05, ^##^ *p* < 0.01, ^###^ *p* < 0.001, ^####^ *p* < 0.0001 versus the MCAO/R + Vehicle group.

Subsequently, in order to clarify whether butyrate improved stroke outcomes in mice, we administered different concentrations of sodium butyrate to mice for 14 consecutive days and then performed MCAO/R. The MCAO/R + Vehicle group had higher neurological deficit scores and infarct volume than the Sham + Vehicle group, indicating that nerve function was seriously damaged, while sodium butyrate administration relatively improved the neurological damage. The administration of 10 mg/kg and 20 mg/kg sodium butyrate declined the neurological deficiency scores and infarct volume in mice; meanwhile, the administration of 20 mg/kg sodium butyrate had the most significant effect (Figure 4A–C). Furthermore, the expression of synapse-related proteins (SYP and PSD95) in MCAO/R + Vehicle mice were decreased versus the Sham + Vehicle group, and the expression of the two proteins was not changed by the pre-administration of 5 mg/kg or 10 mg/kg sodium butyrate. The pre-administration of 20 mg/kg sodium butyrate dramatically upregulated the expression of SYP and PSD95 in the MCAO/R group, suggesting that 20 mg/kg sodium butyrate could improve the synaptic function of model mice (Figure 4D–G). The results of HE and Nissl staining showed that the neurons of the Sham + Vehicle group were intact, with normal nuclei and Nissl bodies, while the neurons in the cerebral ischemic area of the MCAO/R + Vehicle group showed atrophy, vacuolation, nuclear atrophy and Nissl bodies shrinkage. The pre-administration of 5 mg/kg and 10 mg/kg sodium butyrate did not significantly improve the brain injury of model mice, while 20 mg/kg sodium butyrate relatively improved the morphological injury of neurons, indicating that 20 mg/kg sodium butyrate could significantly improve the brain injury of MCAO/R mice (Figure 4H,I). Overall, butyrate was able to improve stroke outcomes in MCAO/R mice with the most pronounced effect at 20 mg/kg of sodium butyrate.

### 3.5. BBR Plays a Neuroprotective Role in the MCAO/R Mice Brain by Promoting the Biosynthesis of Butyric Acid

**Figure 5:**
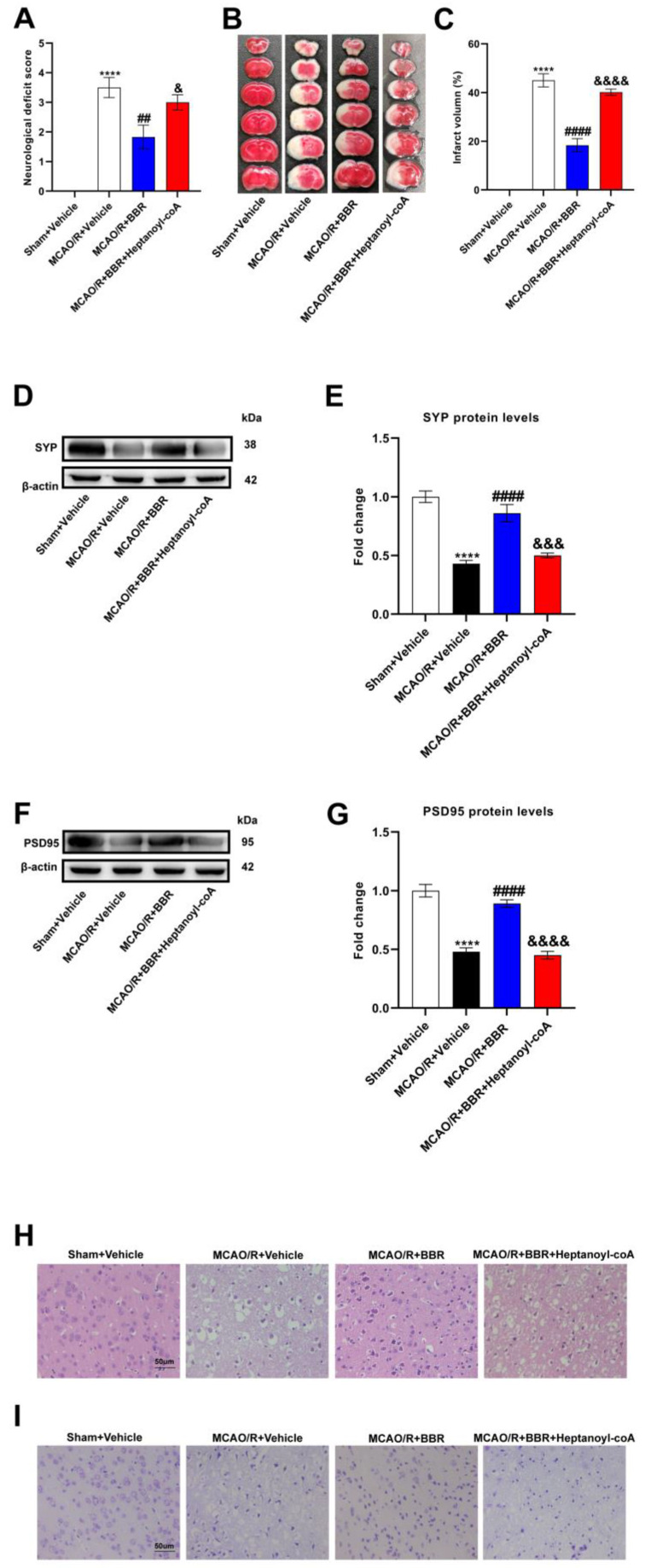
BBR plays a protective role in the MCAO mice brain by promoting butyric acid biosynthesis. (**A**) Neurological scores were measured 24 h after MCAO/R (*n* = 18). (**B**) TTC staining was performed 24 h after MCAO/R (*n* = 6). (**C**) The percentage of infarct volume to Sham value was analyzed 24 h after MCAO/R (*n* = 6). (**D**,**E**) Western blot and quantitative analysis showed the expression of SYP in mice brain (*n* = 6). (**F**,**G**) Western blot and quantitative analysis showed the expression of PSD95 in mice brain (*n* = 6). (**H**,**I**) Representative images of ischemic cerebral tissue with HE staining or Nissl staining (*n* = 6). Scale bar, 50 µm. Results are shown as mean ± SD. **** *p* < 0.0001 versus the Sham + Vehicle group; ^##^ *p* < 0.01, ^####^ *p* < 0.0001 versus the MCAO/R + Vehicle group; ^&^ *p* < 0.05, ^&&&^ *p* < 0.001, ^&&&&^ *p* < 0.0001 versus the MCAO/R + BBR group.

To test whether berberine improves stroke outcomes by promoting butyric acid synthesis, we used a butyric acid synthesis inhibitor, heptanoyl-CoA, which inhibits the key enzyme in butyrate synthesis: butyryl-CoA acetate-CoA transferase (BUT). MCAO/R mice showed higher neurological deficit scores as well as infarct volume, indicating that the nerve function was severely impaired. BBR could significantly reduce the neurological deficiency scores as well as infarct volume of the MCAO/R group, while the inhibitor of butyrate synthesis, heptanyl-CoA, could reverse the above results (Figure 5A–C). Western blot analysis showed that the expression of SYP and PSD95 was strikingly lower in the model mice than the controls, indicating that synaptic function was severely impaired. BBR administration could dramatically increase the content of SYP and PSD 95 and improve the synaptic function, but the administration of heptanoyl-CoA reversed this effect (Figure 5D–G). HE and Nissl staining showed nuclear atrophy, cell membrane vacuolation and Nissl bodies volume reduction in the MCAO/R + Vehicle group. BBR significantly reduced brain injury in the MCAO/R group, but the improvement of BBR for brain damage is inhibited when co-administered with heptanoyl-CoA (Figure 5H,I). Collectively, these results suggested that BBR ameliorates stroke outcomes in mice by enhancing butyrate synthesis.

### 3.6. BBR Inhibits the Level of Pro-Inflammatory Factors in the MCAO/R Mice Brain by Promoting the Biosynthesis of Butyric Acid

**Figure 6:**
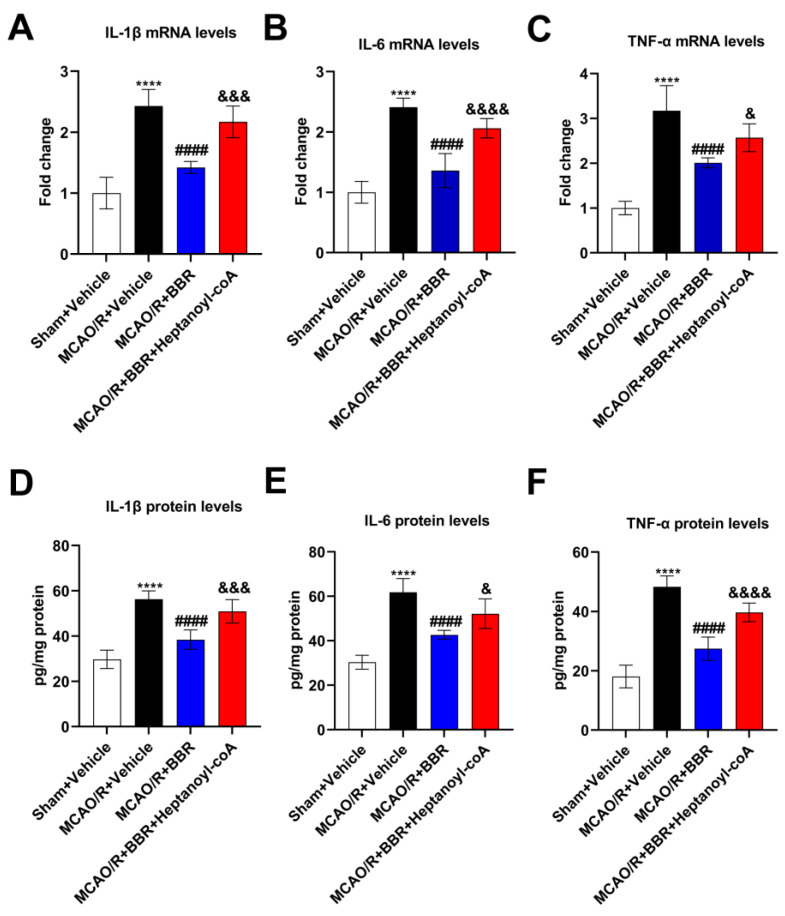
BBR inhibits the level of pro-inflammatory factors in the brain of MCAO/R mice by promoting the biosynthesis of butyric acid. (**A**–**C**) The expression of IL-1β, IL-6, and TNF-α mRNA in the mice brain (*n* = 6). (**D**–**F**) The expression of IL-1β, IL-6 and TNF-α in the mice brain (*n* = 6). β-actin was used as the internal control. Data were presented as mean ± SD. **** *p* < 0.0001 versus the Sham + Vehicle group; ^####^ *p* < 0.0001 versus the MCAO/R + Vehicle group; ^&^ *p* < 0.05, ^&&&^ *p* < 0.001, ^&&&&^ *p* < 0.0001 versus the MCAO/R + BBR group.

Since neuroinflammation is an essential component for the cause of neuronal damage, we examined the levels of inflammatory factors in the mice brain. BBR markedly decreased the mRNA levels of IL-1β, IL-6 and TNF-α in the model mice, and this effect was reversed when BBR was administered together with heptanoyl-CoA, which is an inhibitor of butyrate synthesis (Figure 6A–C). At the same time, ELISA assay proved that the expression of the above three cytokine proteins was remarkably elevated in the MCAO/R + Vehicle mice brains compared with the Sham + Vehicle group. BBR administration could significantly reduce the levels of them, while the co-administration of heptanoyl-CoA and BBR could increase the levels of these three inflammatory factors and thus inhibited the therapeutic effect of BBR (Figure 6D–F). In summary, BBR was shown to promote the synthesis of butyric acid by inhibiting the expression of pro-inflammatory factors in the MCAO/R group.

### 3.7. BBR Inhibits the Activation of Glial Cells and the Expression of NLRP3 by Promoting Butyric Acid Synthesis

**Figure 7:**
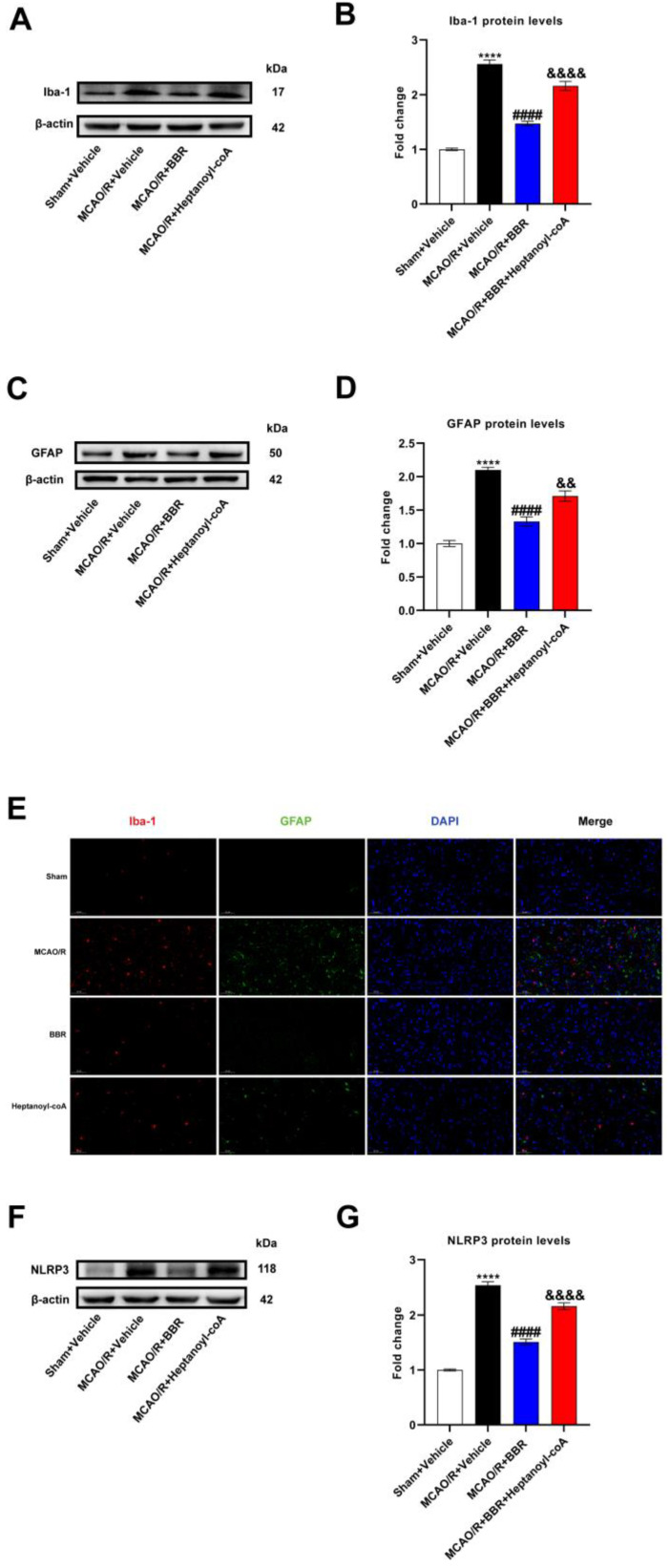
BBR inhibits the activation of glial cells and the expression of NLRP3 by promoting butyric acid synthesis. (**A**,**B**) Western blot and quantitative analysis showed the expression of Iba-1 in mice brain (*n* = 6). (**C**,**D**) Western blot and quantitative analysis showed the expression of GFAP in mice brain (*n* = 6). (**E**) Representative images of immunofluorescence staining for GFAP (green) and Iba1 (red) in ischemic brain tissue of the mice groups (scale bars: 50 μm) (*n* = 6). (**F**,**G**) Western blot and quantitative analysis showed the expression of NLRP3 in mice brain (*n* = 6). Results are presented as mean ± SD. **** *p* < 0.01 versus the Sham + Vehicle group; ^####^ *p* < 0.0001 versus the MCAO/R + Vehicle group; ^&&^ *p* < 0.01, ^&&&&^ *p* < 0.0001 versus the MCAO/R + BBR group.

To additionally confirm the mechanism of neuroprotection of BBR in ischemic stroke, we detected the activation in glial cells in the MCAO/R mice brain after the administration of BBR and heptanoyl-CoA. Western blot results revealed a broad increase in the expression of Iba-1 and GFAP in MCAO/R mice compared to control mice, indicating that microglia and astrocytes were activated in the brains of the MCAO/R group. After BBR administration, the expression of Iba-1 and GFAP was plainly decreased compared with MCAO/R mice, while the co-administration of heptanoyl-CoA and BBR could significantly reverse this effect (Figure 7A–D). We furthermore found via immunofluorescence that the levels of Iba-1 and GFAP in the brain of MCAO/R mice increased significantly versus the control group, and the expression of Iba-1 and GFAP was significantly inhibited after BBR administration, while heptyl-CoA could reverse this effect (Figure 7E). Finally, Western blotting proved a broad increase in NLRP3 expression in the brains of MCAO/R mice compared to control mice. BBR treatment could inhibit the expression of NLRP3, while heptanoyl-CoA reversed this effect of BBR (Figure 7F,G). In summary, BBR is able to suppress the level of NLRP3 as well as the activation of glial cells by promoting butyric acid synthesis.

## 4. Discussion

The brain and gut are connected by neural networks, and there is a two-way interaction, forming a complex gut–brain axis. In particular, the gut microbiome affects this gut–brain two-way communication during the process of ischemic stroke. Ischemic stroke could modify the elements of the gut microbiome. In contrast, the gut microbiota can modulate stroke outcomes and contribute to its progression. It has been demonstrated that the diversity of intestinal flora is decreased in the mice model of ischemic stroke [33]. At the same time, several clinical trials of stroke patients have shown that the gut is dysfunctional after stroke with a reduced abundance of beneficial bacteria (SCFAs-producing bacteria) and an increased abundance of opportunistic pathogens [34,35]. These suggest that intestinal dysbiosis affects the prognosis of IS. In addition, the imbalance of intestinal ecology can further aggravate the brain injury after ischemic stroke. For example, in the mice model of stroke, intestinal dysbiosis increases the number of pro-inflammatory lymphocytes and exacerbates brain damage [36]. Thus, similarly, this study demonstrated that the intestinal microbiota of BBR pretreated mice significantly improved stroke outcomes in MCAO/R mice.

Berberine, an alkaloid derived from Coptis, has received much attention from researchers in recent years. The results of a meta-analysis involving 1670 AIS patients showed that berberine can be used as an adjuvant treatment for AIS by reducing the levels of inflammatory factors [37]. In our study, it was also shown that berberine was finally able to inhibit the release of inflammatory factors to improve stroke. However, berberine is poorly bioavailable and can be enriched in the gut, thus having the ability to regulate gut flora and gut metabolites [38]. One of these studies revealed that the oral administration of berberine in mice modulated the gut flora and increased the number of dopamine-producing bacteria, resulting in increased dopamine production and thus improved the Parkinson’s disease [39]. Another of these studies showed that berberine treatment increased Akkermansia in the intestine and ameliorated atherosclerosis in mice induced by an elevated-fat diet [40]. In this study, we found that BBR could modulate the gut microbiota in MCAO/R mice, increasing the abundance of butyric acid-producing bacteria, including Lachnoclostridium, Clostridioides, Escherichia-Shigella, Bacteroides, Akkermansia, and Klebsiella, and it eventually increased the concentration of butyric acid in mice, resulting in neuroprotective effects in regard to stroke.

Butyric acid is a common metabolite of intestinal probiotics and is an SCFA, which can easily enter the plasma and brain from the gut [41,42]. Numerous studies have shown that butyrate has the functions of alleviating oxidative stress, improving inflammatory response, stimulating gastrointestinal development and enhancing immunity [43,44]. It is worth mentioning that recent studies have also described that berberine acts as a gut microbiome regulator to regulate butyrate production, including in diseases such as arthritis [45] and colon cancer [46]. Recently, butyrate has also been found to have a certain effect on IS, such as reducing ischemia reperfusion injury by reducing neuronal apoptosis and inflammation or promoting angiogenesis in animal models after MCAO [47,48]. Our results showed that the lower abundance of butyric acid-producing bacteria in MCAO/R mice, which may account for the low butyric acid content in MCAO/R mice, and the specific mechanism needs to be further studied.

The development of ischemic stroke is often accompanied by the persistent neuroinflammatory response. As early as the acute phase of IS, microglia in the brain are rapidly activated, while astrocytes also increase in life and become reactive astrocytes [49,50]. The two types of glia migrate to the site of ischemic injury to generate a series of pro-inflammatory cytokines (TNF-α, IL-1β, and IL-6), degrade the extracellular matrix, and further destroy the blood–brain barrier [51]. Neuroinflammatory response is pertaining to the changes in infarct volume induced by ischemic stroke [52]. Blocking the pro-inflammatory activation of glial cells is an effective method to improve ischemic stroke. A study has revealed that butyrate improves alcohol-induced central nervous injury by reducing the abnormal activation of microglia and neuroinflammatory responses [53]. At the same time, butyrate has also proved to play a protective role in reducing the production of inflammatory mediators in vitro models of neuronal injury [54]. Our findings proved that in MCAO/R mice, berberine can promote the biosynthesis of butyrate, which inhibits the abnormal activation and migration of glial cell and reduces the generation of pro-inflammatory factors, thereby reducing the persistent neuroinflammatory response after ischemic stroke.

It should be noted that this study has some limitations. First of all, our study shows a strong link among berberine, the gut microbiota, butyric acid and inflammation. However, immune cells (B or T cells) may play an important role in this process, and we have not studied the function of immune cells. In the course of future studies, we will further explore the link among immune cells, berberine and the gut flora. Second, the decrease in butyrate synthesis in MCAO/R mice may be due to the imbalance of the intestinal flora, which was manifested by the reduction in butyric acid-producing bacteria. However, the underlying mechanism is not investigated and will be further explored in the future.

## 5. Conclusions

In summary, we elucidated the neuroprotection of BBR in IS from the perspective of the gut microbiota, proving that BBR effectively restores the homeostasis of the gut microbiota and promotes the production of butyrate (a bacterial-derived short-chain fatty acid), which further improved the outcome of ischemic stroke by attenuating glial cell-associated neuroinflammation. This study also provides a new experimental basis for the mechanism of berberine in the clinical treatment of IS.

## Data Availability

All data generated or analyzed during this study are available from the corresponding author upon reasonable request.
